# Immunoglobulin γ allotypes influence the level of autoantibody responses to amyloid-β in patients with Alzheimer’s disease

**DOI:** 10.1016/j.humimm.2025.111632

**Published:** 2025-12-09

**Authors:** Janardan P. Pandey, Christine Kimball, Paul J. Nietert, James J. Lah, Allan I. Levey

**Affiliations:** a Department of Pharmacology and Immunology, Medical University of South Carolina, Charleston, SC, USA; b Department of Public Health Sciences, Medical University of South Carolina, Charleston, SC, USA; c Department of Neurology and Goizueta Alzheimer’s Disease Research Center, Emory University School of Medicine, Atlanta, GA, USA

**Keywords:** Alzheimer’s disease, GM/KM allotypes, Autoimmunity, Amyloid-β

## Abstract

Immunoglobulin GM (γ marker) allotypes, highly polymorphic hereditary antigenic determinants of IgG, have been shown to be a risk factor for the development of Alzheimer’s disease (AD). The mechanisms underlying the GM-AD association are not understood. The aim of the present investigation was to determine whether GM genotypes influenced the level of naturally occurring antibodies to amyloid-β (Aβ), a hallmark of AD. We genotyped 100 AD cases and 100 controls for several GM alleles and measured antibodies to Aβ. Results showed that IgG1 GM 3/17 and IgG2 GM 23 genotypes were significantly associated with anti-Aβ antibody levels in AD cases (p = 0.014, 0.018, respectively), but not in controls (p = 0.62, 0.08, respectively). These results, for the first time, show GM allotype restriction in naturally occurring antibody responses to Aβ in individuals with AD. If confirmed, they could help devise a more potent Aβ-based immunotherapy against this neurogenerative disorder.

## 1. Introduction

Immunoglobulin GM (γ marker) allotypes, genetic markers of IgG, have been shown to be a risk factor for the development of Alzheimer’s disease (AD), the most common cause of dementia in older adults [1]. The mechanisms underlying this association are incompletely understood. GM allotypes are known to contribute to antibody responses to a variety of self and nonself antigens [2]. Therefore, mechanisms underpinning their involvement in AD pathogenesis likely include antibody responses to the brain antigens implicated in AD pathology, such as amyloid-β (Aβ) peptide. Naturally occurring anti-Aβ autoantibodies have been shown to contribute to the reduction of Aβ plaque burden [3]. Based on these and other such observations, results from the mouse models of AD, and encouraging results from phase 3 clinical trials, monoclonal anti-Aβ antibodies have been approved for AD therapy. These antibodies are of IgG1 subclass and they have been humanized to reduce immunogenicity. Although this therapy has shown evidence of slowed clinical decline, it is associated with serious side effects (especially in patients homozygous for the *APOE e4* allele), and there is urgent need for more efficacious therapies [4,5]. Understanding the relationship between the genetic risk factors for AD and the brain proteins that are hallmarks of the disease could help design efficacious therapeutic approaches against this devastating neurodegenerative disorder.

The aim of the present investigation was to determine whether GM and KM (κ marker) genotypes influenced the level of naturally occurring antibodies to Aβ in individuals with AD. Analogous to GM, immunoglobulin KM allotypes are also associated with immunity to several self and nonself antigens [2,6].

## 2. Materials and methods

### 2.1. DNA and plasma samples

Coded DNA and plasma samples from AD cases and controls were obtained from the Goizueta Alzheimer’s Disease Research Center Biorepository at Emory University. All participants providing blood samples gave their informed consent following the protocols approved by the Institutional Review Board at Emory University. Clinical diagnoses of AD and control were assigned by consensus conference review of the Uniform Data Set version 3 [7]. Clinical diagnoses were supported by CSF AD biomarkers that were available for 142/200 of the participants. CSF concentrations of Aβ42, t-tau, and p-tau181 were quantified using the first-generation Elecsys ElectroChemiLuminescense Immunoassay (ECLIA) platform on a cobas e 601 analyzer (F. Hoffman-La Roche Ltd).

### 2.2. Anti-Aβ42 antibody determinations

IgG antibody levels to Aβ42 were determined by an enzyme-linked immunosorbent assay (ELISA), using plasma from AD cases and controls. Ninety-six well microtiter plates were coated with the Aβ42 antigen (StressMarq Biosciences, Victoria, British Columbia), 1 μg/ml in 100 μl phosphate-buffered saline (PBS), pH 7.4. After overnight incubation at 4 °C, plates were washed with PBS containing 0.05 % tween 20 (PBS-T) and blocked with PBS-T containing 1 % bovine serum albumin (BSA) for 1 h at 37 °C. Plates were then washed and incubated with diluted (1:100) patient plasma in duplicate wells. Plates were further washed and incubated with anti-human IgG HRP conjugate for 30 min. Finally, plates were washed and incubated with HRP substrate hydrogen peroxide along with 3,3′,5,5′-Tetramethylbenzidine as chromogenic substrate in citrate phosphate buffer, pH 5.5. Reaction was stopped after 20 min by the addition of 100 μl of 2 N HCl and the absorbance values at 450 nm were monitored in a BioTek ELISA reader. Wells containing plasma diluent buffer alone were used as blank. Absorbance values of blank wells were subtracted from the sample wells. Antibodies to Aβ42 were expressed as arbitrary units per microliter (AU/μL), after multiplying with the dilution factor. Anti-Aβ42 antibody determinations were done blinded to the case/control status of the samples.

### 2.3. Genotyping

IgG1 markers GM 3 and GM 17 and IgG2 markers GM 23− and 23 + were previously determined by a TaqMan^®^ genotyping assay [8]. IgG3 allotypes GM 5 and GM 21 were previously genotyped by a polymerase chain reaction-restriction fragment length polymorphism (PCR-RFLP) method [9]. The κchain determinants KM 1 and 3 were previously characterized by a PCR-RFLP technique [10]. *APOE* genotyping was performed previously using an Affymetrix Precision Medicine Array. Genotyping was also done blinded to the case/control status of the samples.

### 2.4. Statistical analysis

T-tests, Wilcoxon rank sum tests, and Fisher’s exact tests were used to compare the distribution of demographic characteristics, genotypes, and antibody levels between cases and controls. Since anti-Aβ42 antibody and the CSF Aβ42 levels were not normally distributed, logarithmic transformations (base 10) were used for statistical analyses. To determine the association of genotypes with the level of anti-Aβ42 antibodies, general linear models (GLMs), which adjusted for age and sex, were constructed. We used an additive model for the genotypes, based on the number of copies of the minor allele, except for the *APOE* gene, in which the number of copies of the *e4* allele—the strongest known genetic risk factor for AD—was used. For all analyses, p < 0.05 was considered statistically significant. No corrections for multiple comparisons were made, due to the hypothesis generating/exploratory nature of the study [11].

## 3. Results

**Fig. 1.**
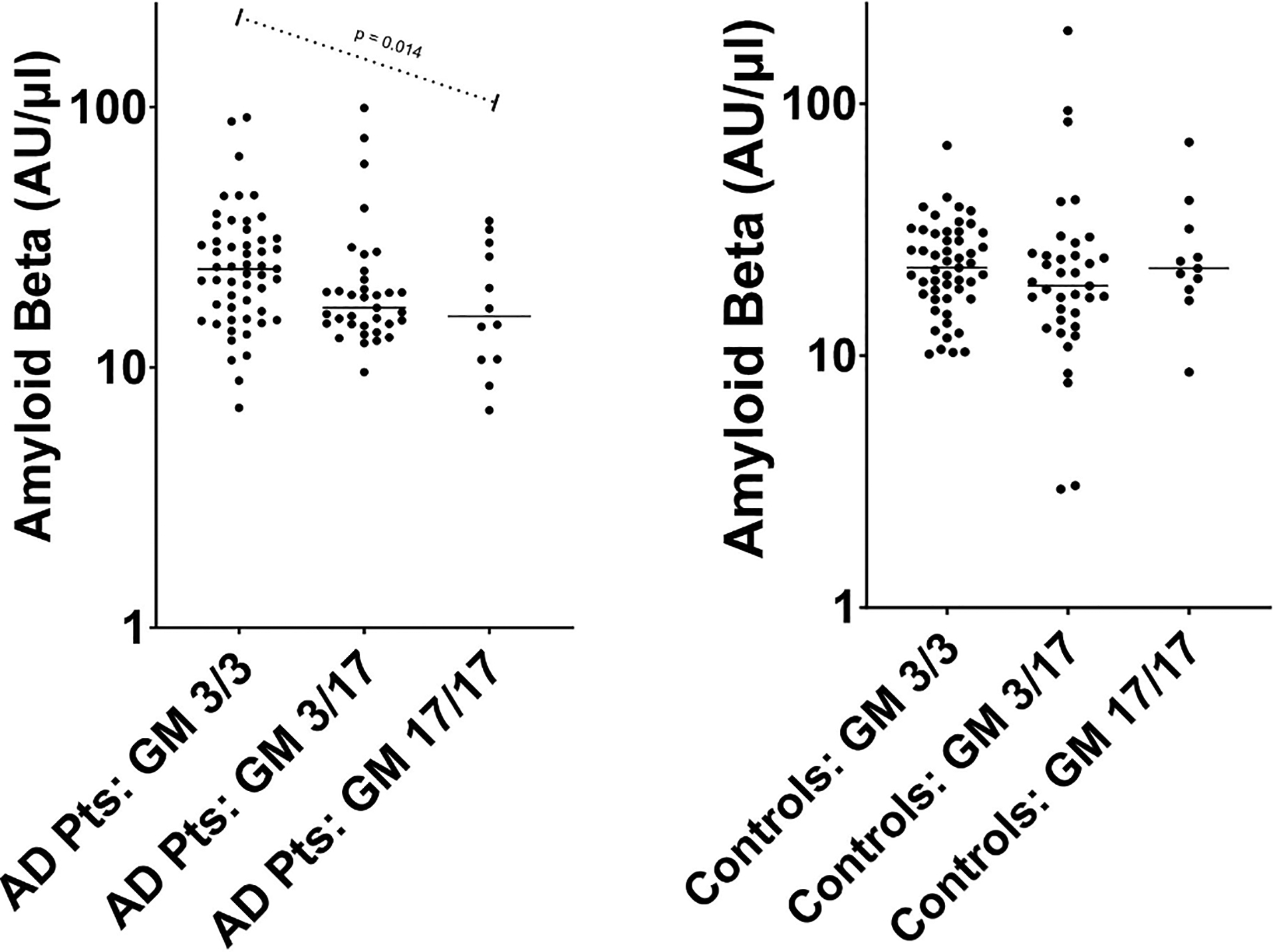
Relationship between GM 3/17 genotype and Amyloid Beta antibody levels. Each individual dot represents an individual study patient (either AD patient [Panel A] or control [Panel B]). Horizontal bars depict means. The reported p-value (p = 0.014) in Panel A was obtained from a general linear model, in which the log_10_ amyloid beta level was expressed as a linear function of the additive effect of the GM 3/17 genotype and adjusted for age and sex. The additive effect of GM 3/17 was not statistically significant (p = 0.62) in the Controls (Panel B).

**Fig. 2.**
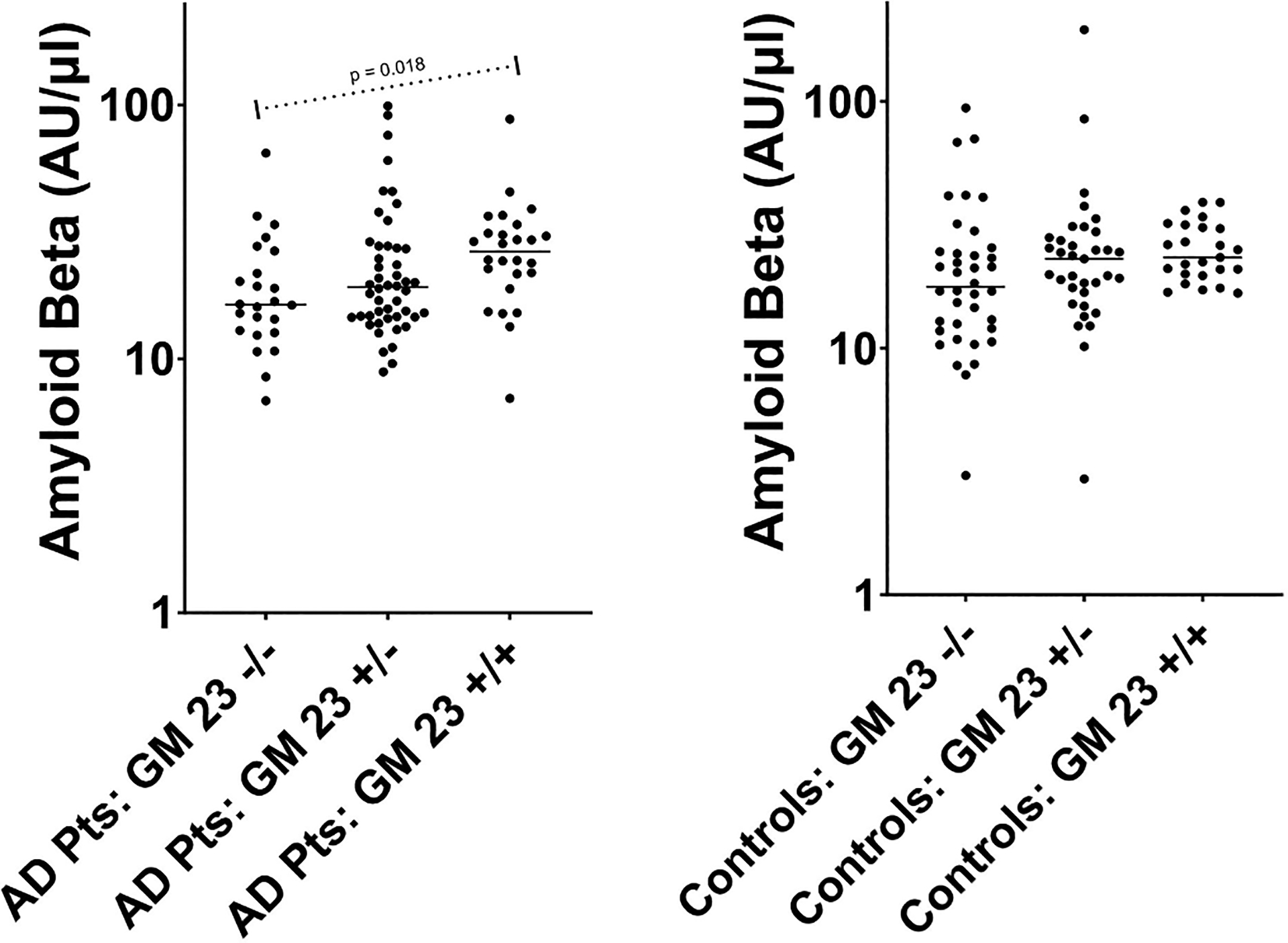
Relationship between GM 23 genotype and Amyloid Beta antibody levels. Each individual dot represents an individual study patient (either AD patient [Panel A] or control [Panel B]). Horizontal bars depict means. The reported p-value (p = 0.018) in Panel A was obtained from a general linear model, in which the log_10_ amyloid beta level was expressed as a linear function of the additive effect of the GM 23 genotype and adjusted for age and sex. The additive effect of GM 23 was not statistically significant (p = 0.08) in the Controls (Panel B).

Table 1 presents the demographic and genotype characteristics of the study population, as well as the plasma anti-Aβ42 antibody levels and the CSF phosphorylated Tau, total Tau, and Aβ42 levels, in AD cases and controls. There were no significant differences between the two groups’ demographics or distributions of the GM or KM genes. There were, however, significant differences in the distribution of *APOE* genotypes and in each of the 3 CSF peptides. As expected, the frequency of *APOE* e4 allele was significantly (p < 0.0001) higher in AD cases compared to controls. Mean CSF phosphorylated Tau and total Tau levels (available for n = 85 and n = 57 of the AD cases and controls, respectively) were significantly (p < 0.0001) higher in AD cases compared to controls, while mean CSF Aβ42 levels were significantly (p < 0.0001) lower in AD cases compared to controls. GM 3/17 genotypes were significantly associated with anti-Aβ42 antibody levels in AD cases (p = 0.014) (Fig. 1, panel A), but not in controls (p = 0.62) (panel B). Among the AD cases, the mean anti-Aβ42 antibody levels decreased with increasing copies of the GM 17 allele (GM 17/17: mean [SD] = 19.2 [10.2] AU/μl; GM 3/17: 23.4 [18.8]; GM 3/3: 27.2 [16.6]). Similarly, GM 23 genotypes were significantly associated with anti-Aβ42 antibody levels in AD cases (p = 0.018) (Fig. 2, panel A), but not in controls (p = 0.08) (panel B). Among the AD cases, the mean anti-Aβ42 antibody levels increased with increasing copies of the “+” GM 23 allele (GM 23 −/−: mean [SD] = 20.2 [12.3] AU/μl; GM 23 +/−: 25.6 [19.7]; GM 23 +/+: 28.4 [15.0]). No other significant associations between the genes of interest and anti-Aβ42 antibody levels were noted.

## 4. Discussion

The results presented here show a strong association between high levels of naturally occurring antibodies to Aβ42 with the homozygosity of GM 3 and GM 23 alleles, which are in significant linkage disequilibrium in Caucasians. It is noteworthy that these associations were detected in AD cases, but not in controls. It is possible that anti-Aβ42 antibodies in individuals with AD are qualitatively different—possibly recognizing different epitopes of Aβ42 from those recognized by the control subjects—and subject to GM allotype restriction.

One mechanism through which GM 3 and GM 23 alleles could influence antibody responses to Aβ42 could be through the antigen processing/presenting pathway. Amino acid substitutions characterizing these alleles—arginine and methionine, respectively—could act as recognition structures/receptors for the relevant Aβ42 epitopes on the membrane-bound IgG of memory B cells. IgG molecules expressing these alleles might be higher affinity receptors than those expressing the alternate alleles, and more efficient in the uptake of Aβ42 and presenting it to helper T cells, resulting in higher B-cell activation.

GM 3 and GM 23 alleles could also influence antibody affinity and specificity by causing conformational changes in the variable regions of γ chains associated with humoral immunity to Aβ42. It is relevant to note that amino acids in the CH1 domain of the γ1 chain—where GM 3 is located—have been shown to modulate the kinetic competence of antigen binding sites [12]. GM 3 and GM 23 alleles could also influence the constitution of the variable-region idiotypes associated with immunity to Aβ42. Although these alleles are expressed in the constant region of the γ chain, they could nonetheless contribute to the conformational changes in the variable region associated with Aβ42 immunity. The contribution of both variable- and constant-region genes to the affinity and specificity of antibodies is well documented [13–15].

It is also possible that there is another as-yet-unidentified locus on chromosome 14 which is the primary determinant of immune responsiveness to Aβ42, and that its alleles are in strong linkage disequilibrium with GM 3 and GM 23 alleles. This could bring about the associations observed in this investigation.

As noted earlier, passive Aβ-based immunotherapy has been approved for early stage AD; however, this therapy has only modest effects on disease progression and yet is associated with serious side effects. An Aβ vaccine that engages the patient’s own immune system would be ideal to provide a sustained anti-Aβ immunity using immunological memory. However, no licensed Aβ vaccine is yet available. Although several Aβ-based vaccine trials have failed, the effort is continuing [16,17]. Understanding the immunogenetic mechanisms underpinning the interindividual variation in humoral immunity to Aβ will be helpful in designing efficient Aβ-based vaccines for AD. Also, if confirmed in a larger study population, these findings could be crucial in the proper evaluation of Aβ-based vaccine (when available) efficacy trials. For instance, some people could be inherently/allotypically high responders to Aβ epitopes, while others could be low responders. This possibility, unless included in the data analyses as a covariate, could confound the evaluation of vaccine efficacy trials.

The present report is the first documenting the role of GM allotypes in immunity to Aβ. It needs to be replicated in an independent investigation. This study has some limitations. The sample size was relatively small (200 subjects) and the study population included white/Caucasian participants only. Given the stark racial/ethnic differences in GM gene frequencies [2], the findings may not be generalizable to other groups.

## Figures and Tables

**Table 1:** Demographic and genotype characteristics for AD cases and controls.

Variable	AD Cases	Controls
	**(N = 100)**	**(N = 100)**
Age at plasma collection (years), mean ± SD	74.2 ± 5.6	74.2 ± 5.6
Sex: n (%) male	40 (40.0 %)	40 (40.0 %)
Sex: n (%) female	60 (60.0 %)	60 (60.0 %)
GM 3/17		
GM 17/17	12 (12.0 %)	11 (11.1 %)
GM 3/17	35 (35.0 %)	37 (37.4 %)
GM 3/3	53 (53.0 %)	51 (51.5 %)
GM 23		
GM 23 +/+	26 (26.5 %)	25 (25.5 %)
GM 23 +/−	48 (49.0 %)	35 (35.7 %)
GM 23 −/−	24 (24.5 %)	38 (38.8 %)
GM 5/21		
GM 21/21	10 (10.1 %)	12 (12.1 %)
GM 5/21	35 (35.4 %)	34 (34.3 %)
GM 5/5	54 (54.5 %)	53 (53.5 %)
KM 1/3		
KM 1/1	3 (3.0 %)	0 (0.0 %)
KM 1/3	14 (14.0 %)	16 (16.2 %)
KM 3/3	83 (83.0 %)	83 (83.8 %)
APOE	*	*
e3/e2	0 (0.0 %)	8 (8.1 %)
e3/e3	25 (28.4 %)	59 (59.6 %)
e3/e4	42 (47.7 %)	25 (25.3 %)
e4/e2	3 (3.4 %)	3 (3.0 %)
e4/e4	18 (20.5 %)	4 (4.0 %)
Anti-Aβ42 IgG (AU/μl), mean ± SD	24.9 ± 16.9	25.7 ± 22.3
Anti-Aβ42 IgG (AU/μl), median (IQR)	19.8 (14.8–29.0)	21.3 (16.6–28.3)
CSF phosphorylated Tau (pg/ml), mean ± SD	44.1 ± 24.9*	18.4 ± 7.8*
CSF phosphorylated Tau (pg/ml), median (IQR)	36.6 (27–54.5) *	17.2 (13.6–21.2) *
CSF total Tau (pg/ml), mean ± SD	353.1 ± 223*	204.5 ± 85.9*
CSF total Tau (pg/ml), median (IQR)	311.3 (241.6–402.1) *	200.1 (155.9–234.8)*
CSF Amyloid β42 (pg/ml), mean ± SD	471.3 ± 212.2*	1172.7 ± 516.9*
CSF Amyloid β42 (pg/ml), median (IQR)	436.9 (308.5 – 624.2) *	1140.0 (781.4 – 1500.0) *

* P < 0.0001 by Fisher’s exact test or independent samples T-test, as appropriate.

## Abbreviations:

GM: γ marker
AD: Alzheimer’s Disease
Aβ: amyloid-β
KM: k marker
ELISA: enzyme-linked immunosorbent assay
PBS: phosphate-buffered saline
PCR-RFLP: polymerase chain reaction-restriction fragment length polymorphism method
GLM: general linear model
